# Notes and News

**Published:** 1889-12-07

**Authors:** 


### Notes and News.

Collected and Communicated.

Amongst the many wordy and often hysterical appeals we receive from charities, one at last has reached us from the Homes for Working Boys in London, which is simple and concise, and appeals, therefore, very forcibly to business men. It runs as follows: "Dear Sir,—Will you do me the favour of reading the enclosed, which I think you will find of interest. The homes are doing very well, but we are in need of funds.—Yours faithfully, T. H. W. Pelham."

Here is that extremely wicked North-West London Hospital issuing a list of 300 prizes of which you can take your chance by subscribing 10s. to its funds. Now if these prizes were fat pigs and steam rollers of the usual lottery order it wouldn't matter; but the list published is so enticing it is almost impossible not to join in this charity gamble. There are cigars, easy-chairs, portraits of Ellen Terry, complete works of Lord Tennyson in seven vols. on hand-made paper, and other articles that would bribe even Andrew Lang. But what will happen if a gentleman wins prize 118, the gift of Madame Esmeralda, and "made to order"; or if a lady wins 214, the gift of Mr. G. Clark, of Hanover-square, and also "made to order"? We see that Messrs. Burroughes, Welcome, and Co. have given a travellers' medicine case.

The Jubilee wing of Dover Hospital was formally opened on November 21. The proceedings commenced with a service at St. Mary's Church at 11 a.m., when a short dedicatory address was given by the vicar, the Rev. Canon Puckle. The ceremony at the hospital followed immediately after. The Mayor, at the invitation of the Rev. Canon Puckle, addressed the meeting. His worship said among the many duties which it might fall to his lot to discharge during his year of office, there could be none which would appeal more to his sympathies than the task he was asked to perform that day. He was reminded that their present hospital was established as a thankoffering of the inhabitants in 1849, when they felt but slight effects of the cholera which was raging all over England. He also expressed the general feeling of gratitude to Canon Puckle felt by all present. The building was then inspected. Besides various offices, there are three wards for male patients, and three for female patients. Each of these wards, which are 14ft. high and about 24ft. square, when properly furnished will be provided with six or eight beds. A Queen Anne's grate in each throws out an abundance of warmth, but a uniform temperature is still more induced by hot-water pipes running round the room, while in close proximity are two consulting-rooms. The wing is built from designs by Mr. Grant, of Sittingbourne.

Here is a good specimen of local politics. A letter has reached us from Chichester which runs as follows: "As, no doubt, you would surmise, your article in The Hospital of November 16 has much enraged many of the drainage party in this city, and they are now setting about the following plan of revenge. They are collecting all the copies of The Hospital they can together—for they have been freely distributed in the town—for the purpose of returning them to you. They will send them carriage unpaid." Of course, we were amused to receive this warning, and shall act upon it.

The *Globe* is responsible for the following, and if it isn't true it ought to be, it is so good: "An M.P. went down to inspect the asylum of which he was governor, and it happened to be the night of the weekly entertainment for the inmates, and the entertainment happened to be a ball, and the M.P. kindly danced with a fair lunatic. 'I don't remember having seen you here before,' said she; 'how long have you been in the asylum?' 'Oh, I only came down yesterday,' said he, 'as the Parliamentary inspector on the governing body.' 'Of course,' returned she; 'how stupid I am! I knew you were either an inmate or a member of Parliament as soon as I looked at you.'"

A quarterly general court of the governors of the Middlesex Hospital was held on Thursday, the 28th ult., Mr. Henry N. Custance in the chair. Among the governors present were Mr. J. Bell Sedgwick, J.P., deputy-chairman of the Weekly Board; Mr. Francis Hoare, treasurer; Lieut.-Col. Hollway, Mr. Burroughes, Rev. T. Echalaz, Mr. C. C. Cotes, Dr. Finlay, Dr. Pringle, Dr. Becher, and numerous others. The chairman alluded to the satisfactory progress made by the hospital during the past quarter, and hoped that the improvement noticeable in the receipts would continue, but pointed out that this could only result from the claims of charity, especially in regard to the cancer wards, being more widely known. The proceedings closed with the customary vote of thanks to the chairman.

A special court of governors of the Royal Hospital for Diseases of the Chest was held on Thursday of last week to discuss the council's refusal to reconsider the dismissal of the late secretary, Mr. J. J. Austin. The proceedings were lively, but good tempered. The arrangements were unusually bad, seeing that a relatively small and inconvenient room had been selected (though it is but right to say that a larger one was not then available) at the Cannon-street Hotel, where the meeting was held. However, the result was that quite a hundred governors were unable to obtain admission. We were glad to see some of the most influential men of the City of London amongst the audience—a fact of considerable promise for the progress of sound hospital administration in this country. The late Lord Mayor (Sir James Whitehead, Bart.) was elected chairman, on the motion of Lord Charles Bruce, the president of the hospital.

Sir James Whitehead put the facts before the meeting, and suggested that the objecting governors should nominate six of their number to confer with the council. It was proposed that this suggestion should be embodied in a resolution with a vote of confidence in the council itself, but Mr. George Berry, the leader of objectors, declined to accede to any vote of confidence. A resolution of confidence in the council was then moved by Lord Derby, seconded by the president of the College of Surgeons, Mr. Hutchinson, and carried, amidst cheers, by an overwhelming majority. The ex-Lord Mayor's suggestion was then formally moved, but many of the governors considering it unnecessary voted against it, although it was ultimately carried. We heartily congratulate the council on their well-merited triumph.

The following vacancies are announced this week, the amount of the salary in each case being given after the name of the institution:

**Resident Medical Officers.**

York County Hospital. Assistant Surgeon—£40, board, &c.
Miller Hospital and Dispensary. Junior Medical Officer—£30, board, &c.
Monkstown Hospital. Medical Officer—£40, board, &c.

**Non-Resident Medical Officers.**

National Sanatorium for Consumption, Bournemouth. Physician.
St. George's Hospital. Lecturer on Dental Surgery.
King's College Hospital. Assistant Surgeon, Assistant Physician, and Assistant Accoucheur.
North-West London Hospital. Assistant Physician.

---

The annual meeting of the friends of the Manchester and Salford Hospital Sunday and Saturday Funds has been held, and a most satisfactory report adopted. The amount paid to the Hospital Sunday account has been £4,636 10s. 4d. against £4,559 3s. 1d. last year, which shows an increase of £77 7s. 3d.; while the amount contributed to the Hospital Saturday Fund has been £3,536 12s. against £3,432 14s. 10d., or an increase of £103 17s. 2d, the total increase in the contributions for this year being £181 4s. 5d. Mr. G. Rooke said that the medical charities were responsible for a large proportion of the reduction in pauperism which had taken place, and therefore those who subscribed to them might consider the subscriptions an investment from which they could have a return in the reduction of the poor rate.

The Bishop of Shrewsbury presided at the annual meeting of North Staffordshire Infirmary at Hartshill. The annual report stated that the income from all sources had been £11,268, against £9,759 last year, showing an increase of £1,509. The expenditure amounted to £9,418, against £8,753 the previous year, the increase being £665. The total number of patients treated during the year had been 11,018, against 11,293 the previous twelve months. The number of in-patients admitted had been 1,551, against 1,512. The number of childien treated as in-patients was 103, included in the previous number, compared with 111, and the number of out-patients and casualties treated 9,316, against 9,603 the previous year. During the year many of the sanitary appliances, some of them many years old, had been removed and replaced by more modern ones. The additional accommodation for the nurses had been provided, and the alterations were expected to add materially to the usefulness of the institution. The North Staffordshire Convalescent Fund has, as usual, proved a most beneficial adjunct to the hospital.

---

At a late meeting of the Southend Local Board, a Mr. Gosset caused much laughter by an extraordinary account of an interview with Mr. Phillips, medical officer of the Infectious Diseases Hospital. A few words will suffice as an example: "Subsequently he (Mr. Gosset) met Mr. Phillips, when he greeted him something like this: 'Good God, doctor, what have you done?' (laughter). The doctor, with a bland and almost childish smile (laughter) asked him what he meant. He explained to Mr. Phillips that he had signed this report when he was the person who was necessarily and primarily responsible for the state of things referred to (laughter) and also that it was upon him that the blame must eventually fall. Mr. Phillips thereupon expressed his annoyance at having signed the report." After half a column of this, someone was found with the sense to desire the presence of the doctor, Mr. Phillips, to which Mr. Gosset replied he "was merely stating facts." It is a great pity that the chairman of a meeting should not be more careful to exercise his control, and maintain the dignity of the board over which he presides.

Belfast Royal Hospital quite cheerfully reports a deficit of £1,147. The secretary said this was chiefly due to the enormous increase in the number of patients. No fewer than 2,361 had been admitted as intern patients, while no less than 26,581 had been treated as extern patients, making a total of 28,941 persons who had participated in the benefits of the great charity during the past year. A great many minor improvements have been made lately at the hospital, chiefly owing to the public criticism evoked by the Jeffries case. It is hoped that the trustees of the Campbell bequest will consider the merits and needs of Belfast Royal Hospital.

---

On Saturday last the Duchess of Albany visited the Polytechnic and delivered certificates to 117 successful ambulance pupils. Sir V. Kennett Barrington occupied the chair, and delivered a lengthy address, in the course of which he observed that the Duchess of Albany had always shown marked interest in the work, as she had gone through its whole course of classes, and holds its highest nursing certificates, while her own and the Princess of Wales's labours in connection with the Red Cross Society had endeared them personally to every soldier who had been sick or wounded in any of our recent campaigns. Amongst those who received certificates was Mrs. Rider Haggard.

---

The annual meeting of subscribers to the North Lonsdale Hospital was held in the board-room at the hospital on 20th November. A few working-men from some of the large works were present. Sir James Ramsden occupied the chair. The subscriptions during the year amounted to £1,263 9s. 11d., the expenditure being £1,147 3s. 9d. On the 30th June last there was a balance due to the bank of £78 19s. 5d., but this had been wiped out, and the hospital had now a balance at the bank of £37 16s. 5d. The chief point brought forward was the desire of the working-men to be more largely represented on the committee. It was finally agreed to suggest to the council that there should be two representatives from the shipyard, one from the iron works, one from the steel works, one from the railway works, and one from each of the other works.

A quarterly court of the governors of the Hospital for Consumption, Brompton, was held in the board-room of the hospital on the afternoon of December 5, Mr. T. Percival Beckwith in the chair. Among the governors present were: Mr. Robert Gillespie, the Hon. Colonel Talbot, Mr. Arthur Roberts, Mr. Gerald Young, Mr. W. J. Juby, Dr. Symes Thompson, and Dr. Tatham. The report of the Committee of Management, read by the secretary (Mr. Dobbin), stated that since the last court the whole of the older hospital had been placed in the builder's hands, and the needful repairs, etc., having been completed, the 321 beds in the two buildings were again reoccupied. The committee had continued to send patients to the convalescent homes of the London Samaritan Society, where they were maintained at the cost of the hospital, thus extending the field of usefulness of the charity. This arrangement still worked beneficially for the patients, and the committee trusted that the public would afford the increased support which was urgently needed. A grant of £1,718 had been received from the Hospital Sunday Fund. One legacy only had been announced—viz., from Mr. Geo. Stevenson, £100 duty free. The committee referred with deep regret to the death of the Earl of Leven and Melville, a liberal supporter of the charity. The weekly musical and other performances, long a source of much benefit and amusement to the inmates, had been resumed. The number of in-patients admitted since August 1 was 568; discharged, many benefited, 429; died, sixty-nine; new out-patient cases, 4,319. Mr. W. S. Deacon was unanimously elected treasurer in the room of the late Lord Leven, and the proceedings closed by a cordial vote of thanks to the chairman.

